# Construction, validation and, visualization of a web-based nomogram to identify the best candidates for primary tumor resection in advanced cutaneous melanoma patients

**DOI:** 10.3389/fsurg.2022.975690

**Published:** 2023-01-18

**Authors:** Zhehong Li, Junqiang Wei, Honghong Zheng, Yafang Zhang, Yange Zhang, Haiying Cao, Yu Jin

**Affiliations:** ^1^ Traumatology and Orthopaedics, Affiliated Hospital of Chengde Medical University, Chengde, China; ^2^ Department of General Surgery, Beijing Shijitan Hospital, Capital Medical University, Beijing, China; ^3^ General Surgery, Affiliated Hospital of Chengde Medical University, Chengde, China

**Keywords:** cutaneous melanoma, stage IV, surgery, nomogram, SEER database

## Abstract

**Background:**

Existing studies have shown whether primary site resection (PSR) in cutaneous melanoma (CM) patients with stage IV is controversial. Our study aimed to identify the clinical characteristics of CM patients with stage IV who benefited from PSR on a population-based study.

**Methods:**

We retrospectively reviewed stage IV CM patients in the Surveillance, Epidemiology, and End Results (SEER) database from 2004 to 2015. Patients were divided into surgical and non-surgical groups according to whether PSR was performed or not. According to the median cancer-specific survival (CSS) time of the non-surgery group, the surgical group was divided into the surgery-benefit group and the non-surgery-benefit group. Multivariate cox regression analysis was used to explore independent CSS prognostic factors in the surgical group. Then, based on the independent prognostic factors of the surgical group, we established a web-based nomogram based on logistics regression.

**Results:**

A total of 574 stage IV CM patients were included in our study, and 491 (85.60%) patients were included in the surgical group. The clinical characteristics (benefit group and non-benefit group) included age, M stage, lesion location, and ulceration status. These independent prognostic factors were includeed to construct a web-based nomogram.

**Conclusions:**

We constructed a web-based nomogram. This model was suitable for identifying the best candidates suitable for PSR in stage IV CM patients.

## Introduction

Cutaneous melanoma (CM) is a highly aggressive malignant tumor that originates from melanocytes (1). Global Cancer Statistics demonstrated that 324,635 new CM individuals were diagnosed and 57,043 deaths for the disease worldwide in 2020 (2). Although the five-year survival rate for CM patients with stage I-III is high [Five-year cancer-specific survival (CSS) for cutaneous melanoma at stage I, II, and III was 98%, 90%, 77%], the five-year survival rate for stage IV CM patients is less than 20% (3, 4). In the past period, based on further understanding of the molecular pathogenesis of melanoma, significant changes have taken place in the treatment of advanced CM patients. The application of immunotherapy [e.g., checkpoint inhibitors against cytotoxic T lymphocyte antigen 4 (CTLA-4) and/or programmed death 1 (PD-1)], molecular targeted anti-tumor therapy [B-Raf proto-oncogene (BRAF), mitogen-activated protein kinase (MEK)], and neoadjuvant therapy has greatly improved the survival prognosis of CM patients(1, 5–7). However, for stage IV CM patients, the primary site resection (PSR) is controversial because it is a local treatment for a systemic disease (8). Based on the metastatic potential of CM, PSR for stage IV CM is unsatisfactory, and therefore many scholars do not recommend surgery for stage IV CM patients (9, 10). However, another part of the scholars’ research showed that the prognosis of stage IV CM patients could be improved by PSR or metastatic lesions surgery(11–13). Therefore, there is still some controversy about whether patients with stage IV melanoma should perform PSR. PSR in stage IV lung cancer patients is also controversial. However, a recent retrospective study has suggested that stage IV lung cancer patients with specific clinicopathological features may benefit from PSR (14). Inspired by these conclusions, we also came up with a new idea that not all stage IV CM patients will benefit from PSR, and patients with specific characteristics can benefit from PSR.

However, large-scale population-based studies are still lacking, and it is clinically significant to screen for the types of patients who would benefit from PSR. Therefore, we aimed to analyze the stage IV CM patients in the Surveillance, Epidemiology, and End Results (SEER) database and establish a web-based nomogram to identify the best candidates for PSR and their characteristics.

## Methods

### Patients

We obtained permission to access these study data (15708-Nov2020). The inclusion criteria were: patients diagnosed with stage IV CM between 2004 and 2015 with complete follow-up data. Exclusion criteria were as follows: age less than 18 years, Race unknown, TNM stage unknown, treatment unknown, mitotic status unknown, not the first tumor. We obtained baseline data from the SEER database, including patient information (age, sex, and race), melanoma characteristics TNM stage (AJCC 7th Edition Melanoma), location, histological type, mitotic rate, and ulceration), and surgery (primary site resection). CSS was defined as the time from diagnosis to death because of the CM. According to whether the PSR was performed or not, Patients were divided into surgical and non-surgical groups. Based on the median CSS time (8 months) of the non-surgical group, we divided the surgical sets into the surgical beneficial and the surgical non-profitable groups.

### Statistical analysis

We used t-tests and chi-square tests for comparing continuous and categorical variables, respectively. Multivariate Cox regression analysis was performed to identify independent prognostic factors associated with CSS. Hazard Ratios (HRs) with 95% confidence intervals (CIs) for each factor were calculated. Statistical analyses were performed by R software (version 4.0.3), all statistical tests were two-sided, and *p*-value <0.05 was considered statistically significant.

### Construction, validation, and visualization of a web-based nomogram

Zhang et al. have demonstrated that patients with PSR have a longer median CSS time than those who did not undergo surgery (13). Based on this conclusion, patients who underwent PSR were randomly divided 7:3 into training and validation sets by the “caret” package. We build a logistics-model nomogram based on the independent prognostic factors of CSS. We use the area under the curve (AUC) of the receiver operating characteristic curve (ROC), the calibration curve, and the decision curve analysis (DCA) to evaluate the discriminative ability and accuracy of the nomogram both in training and validation sets. Then, a web-based nomogram was performed using the “Dynnom” package. Finally, based on the results of our prediction model, we divided all patients into three groups, the surgery & beneficial group (probability of benefit >50%), the surgery & non-beneficial group (probability of benefit <50%), and the non-surgical group. The patients in the three groups were analyzed by Kaplan-Meier (K-M), and log-rank tests were calculated. All statistical analyses and image visualizations were performed using R software (version 4.0.3).

## Results

### Patients clinicopathological characteristics

**Figure 1:**
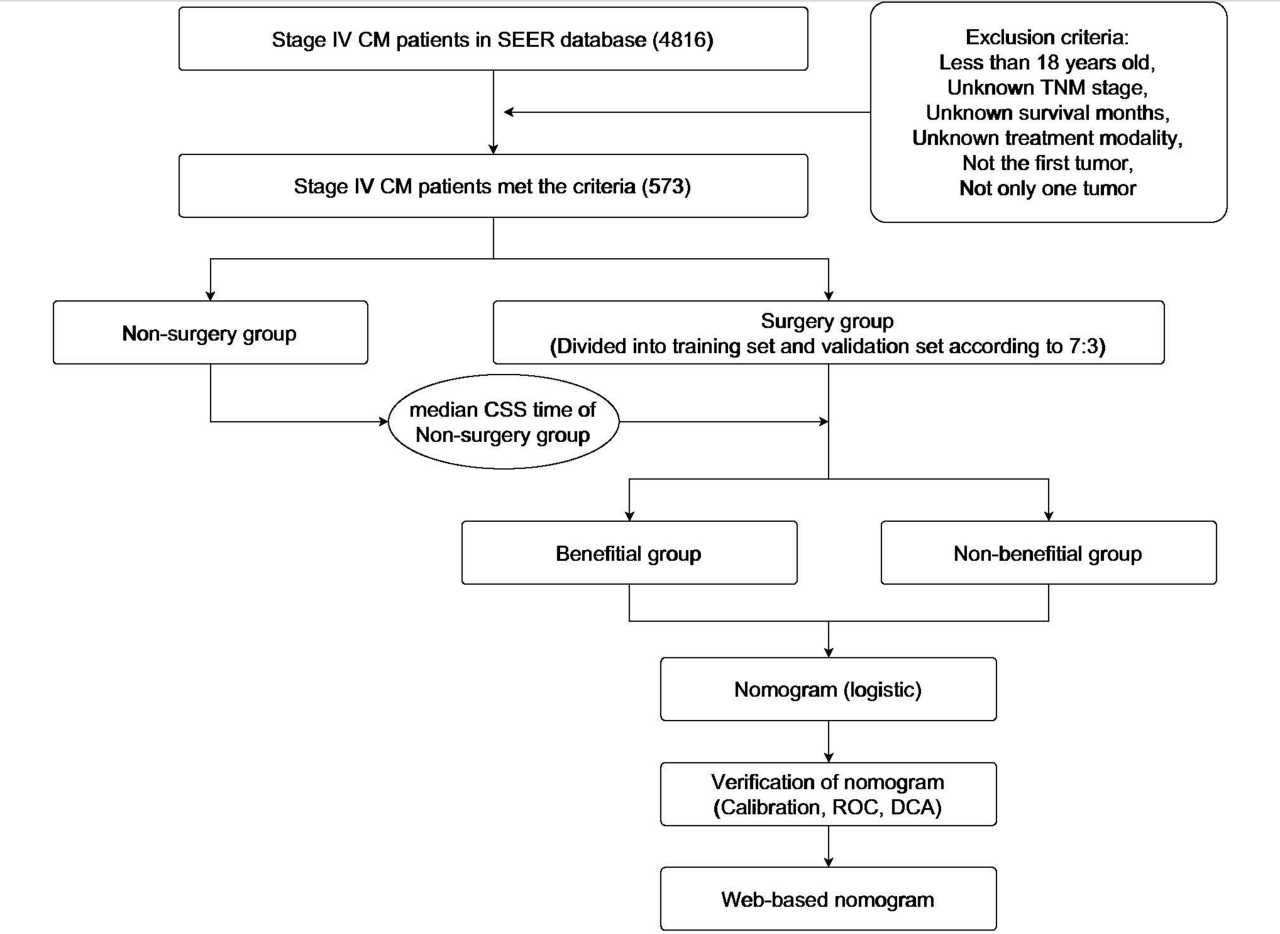
The design idea and workflow.

We identified 573 patients with stage IV CM who met the criteria from the SEER database (see Figure 1). Of these eligible patients, 491 (85.69%) received PSR. Through t-test for age, chi-square test for sex, race, location, histological type, TNM stage, ulceration, and mitosis rate. Patients' clinicopathological data in the surgical and non-surgical groups were relatively balanced (*p* > 0.05). The results showed that the clinicopathological characteristics of the two sets (surgical and non-surgical groups) were comparable (Table 1).

**Table 1:** Demographic information for stage IV melanoma patients.

	All patients	Surgery to primary site (*n*, %)		Non-surgery to primary site (*n*, %)		*χ*²/*t*	*p*-value
Age						0.924	0.356
Mean	63.07	64.52		62.85			
SD	15.17	13.47		15.43			
Race						0.602	0.740
White	549	470	95.72	79	96.34		
Black	13	12	2.44	1	1.22		
Other	11	9	1.83	2	2.44		
Sex						0.304	0.581
Female	167	141	28.72	26	31.71		
Male	406	350	71.28	56	68.29		
Location						1.959	0.743
Head and neck	150	132	26.88	18	21.95		
Trunk	209	179	36.46	30	36.59		
Upper limb and shoulder	87	75	15.27	12	14.63		
Lower limb and hip	111	91	18.53	20	24.39		
Others	16	14	2.85	2	2.44		
Subtype of melanoma						19.128	<0.001
Malignant melanoma	263	208	42.36	55	67.07		
Nodular	185	173	35.23	12	14.63		
Superficial spreading	50	44	8.96	6	7.32		
Others	75	66	13.44	9	10.98		
T stage						1.908	0.592
T1	87	71	14.46	16	19.51		
T2	88	74	15.07	14	17.07		
T3	112	98	19.96	14	17.07		
T4	286	248	50.51	38	46.34		
N stage						0.887	0.829
N0	227	195	39.71	32	39.02		
N1	160	136	27.70	24	29.27		
N2	71	59	12.02	12	14.63		
N3	115	101	20.57	14	17.07		
M stage						6.332	0.042
M1a	125	110	22.40	15	18.29		
M1b	103	95	19.35	8	9.76		
M1c	345	286	58.25	59	71.95		
Ulceration						0.827	0.363
No	178	149	30.35	29	35.37		
Yes	395	342	69.65	53	64.63		
Mitotic rate						4.083	0.130
<1	436	367	74.75	69	84.15		
=1	100	92	18.74	8	9.76		
≥2	37	32	6.52	5	6.10		
Radiation						3.542	0.060
No	400	350	71.28	50	60.98		
Yes	173	141	28.72	32	39.02		
Chemotherapy						0.446	0.504
No	409	353	71.89	56	68.29		
Yes	164	138	28.11	26	31.71		

### Independent risk factors for CSS in stage Iv Cm patients with primary tumor resection

**Figure 2:**
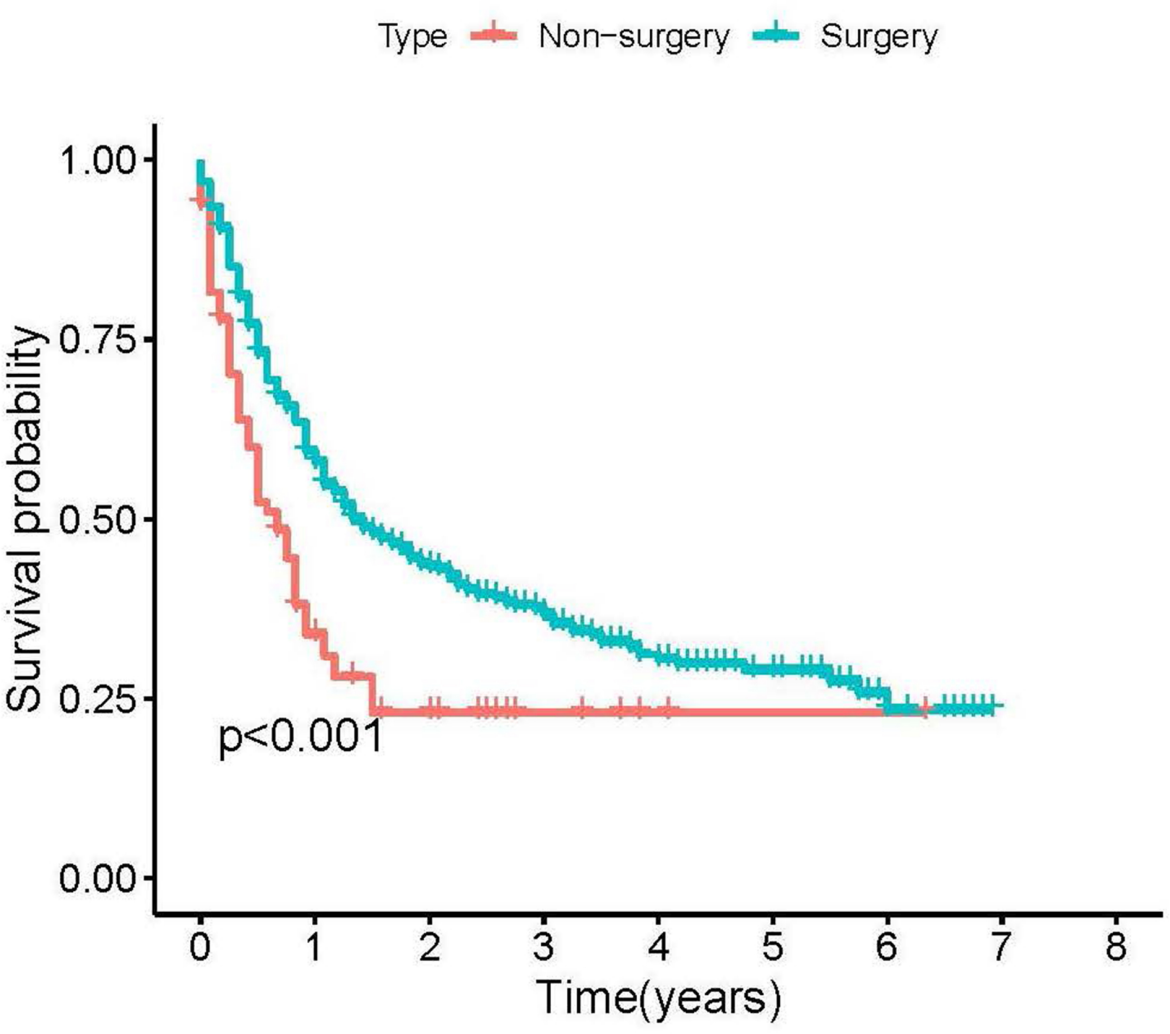
Kaplan-Meier plots of stage IV cutaneous melanoma patients according to primary site resection for cancer-specific survival.

The median CSS time in the surgical group was 17 months (95%CI = 13.603–20.397 months), and the median CSS in the non-surgical group was 8 months (95%CI = 5.142–10.858 months). The K-M analysis and log-rank test of the surgical and the non-surgical groups are shown in Figure 2. The results show that patients with PSR can benefit more than patients without PSR. Then, patients in the surgical group were further divided into the training set (*n* = 347, 70.67%) and the validation set (*n* = 144, 29.33%). Comparability of the training and validation sets was confirmed by the t-test and chi-square test (see Table 2). Multivariate Cox regression analysis on the surgical group, age, M stage, lesion location, and ulceration status were independent prognoses for CSS (see Table 3).

**Table 2:** Clinicopathological characteristics of training set and validation set.

	Training set (*n*, %)		Validation set (*n*, %)		χ²/*t*	*p*-value
Age					1.315	0.189
Mean	63.44		61.43			
SD	15.21		15.93			
Race					0.986	0.611
White	334	96.25	136	94.44		
Black	7	2.02	5	3.47		
Other	6	1.73	3	2.08		
Sex					0.088	0.767
Female	101	29.11	40	27.78		
Male	246	70.89	104	72.22		
Location					3.490	0.479
Head and neck	93	26.80	39	27.08		
Trunk	126	36.31	53	36.81		
Upper limb and shoulder	52	14.99	23	15.97		
Lower limb and hip	63	18.16	28	19.44		
Others	13	3.75	1	0.69		
Subtype of melanoma					2.384	0.497
Malignant melanoma	145	41.79	63	43.75		
Nodular	118	34.01	55	38.19		
Superficial spreading	33	9.51	11	7.64		
Others	51	14.70	15	10.42		
T stage					2.532	0.47
T1	53	15.27	18	12.50		
T2	50	14.41	24	16.67		
T3	74	21.33	24	16.67		
T4	170	48.99	78	54.17		
N stage					6.366	0.095
N0	149	42.94	46	31.94		
N1	93	26.80	43	29.86		
N2	36	10.37	23	15.97		
N3	69	19.88	32	22.22		
M stage					0.470	0.791
M1a	75	21.61	35	24.31		
M1b	67	19.31	28	19.44		
M1c	205	59.08	81	56.25		
Ulceration					0.023	0.88
No	106	30.55	43	29.86		
Yes	241	69.45	101	70.14		
Mitotic rate					0.449	0.799
<1	260	74.93	107	74.31		
=1	66	19.02	26	18.06		
≥2	21	6.05	11	7.64		

**Table 3:** Multivariate Cox analysis for CSS among population of surgery to primary site.

	Adjust HR	95%CI	*p*-value
Age	1.011	1.001–1.021	0.027
Race
White	Reference		
Black	1.57	0.703–3.507	0.271
Other	1.761	0.604–5.319	0.300
Sex
Female	Reference		
Male	1.033	0.751–1.419	0.843
Location
Head and neck	Reference		
Trunk	1.675	1.144–2.451	0.008
Upper limb and shoulder	1.683	1.064–2.660	0.026
Lower limb and hip	1.535	0.959–2.458	0.074
Others	1.736	0.851–3.540	0.129
Subtype of melanoma
Malignant melanoma	Reference		
Nodular	1.055	0.755–1.475	0.754
Superficial spreading	1.234	0.729–2.087	0.434
Others	1.074	0.697–1.654	0.746
T stage
T1	Reference		
T2	0.892	0.521–1.528	0.678
T3	0.787	0.479–1.294	0.345
T4	0.863	0.547–1.362	0.528
N stage
N0	Reference		
N1	0.875	0.608–1.258	0.47
N2	0.745	0.436–1.274	0.282
N3	1.088	0.736–1.610	0.672
M stage
M1a	Reference		
M1b	1.671	0.980–2.851	0.059
M1c	3.694	2.361–5.779	<0.001
Ulceration
No	Reference		
Yes	1.441	1.030–2.014	0.033
Mitotic rate
<1	Reference		
=1	1.053	0.733–1.513	0.78
≥2	0.829	0.427–1.610	0.58

### Establishment and visualization of the nomogram

**Figure 3:**
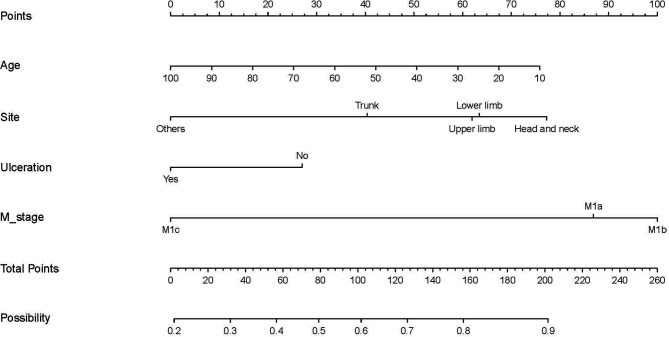
A nomogram to predict optimal candidates for primary tumor resection.

We defined that a patient who underwent PSR benefited if the survival time exceeded the median CSS time without surgery (8 months). Therefore, patients in the surgery group with survival times longer than 8 months were defined as the surgery benefit group; those with less than or equal to 8 months were defined as the surgery non-benefit group. Independent prognostic factors (age, M stage, lesion location, and ulceration status) were included in the logistics regression model to establish a nomogram in the training set (see Figure 3).

### Validation of nomogram and establishment of the web-based nomogram

**Figure 4:**
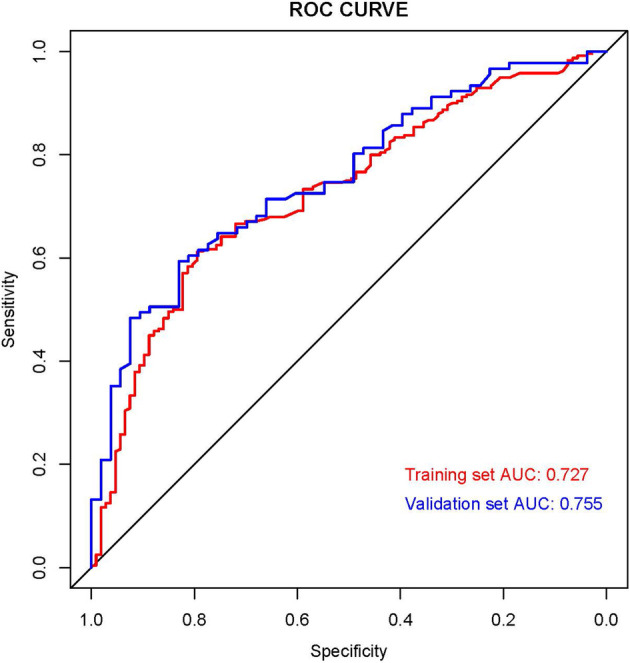
ROC curves of the nomogram. ROC curves of the nomogram in the training set (Red) and validation (Blue). ROC, receiver operating characteristic.

**Figure 5:**
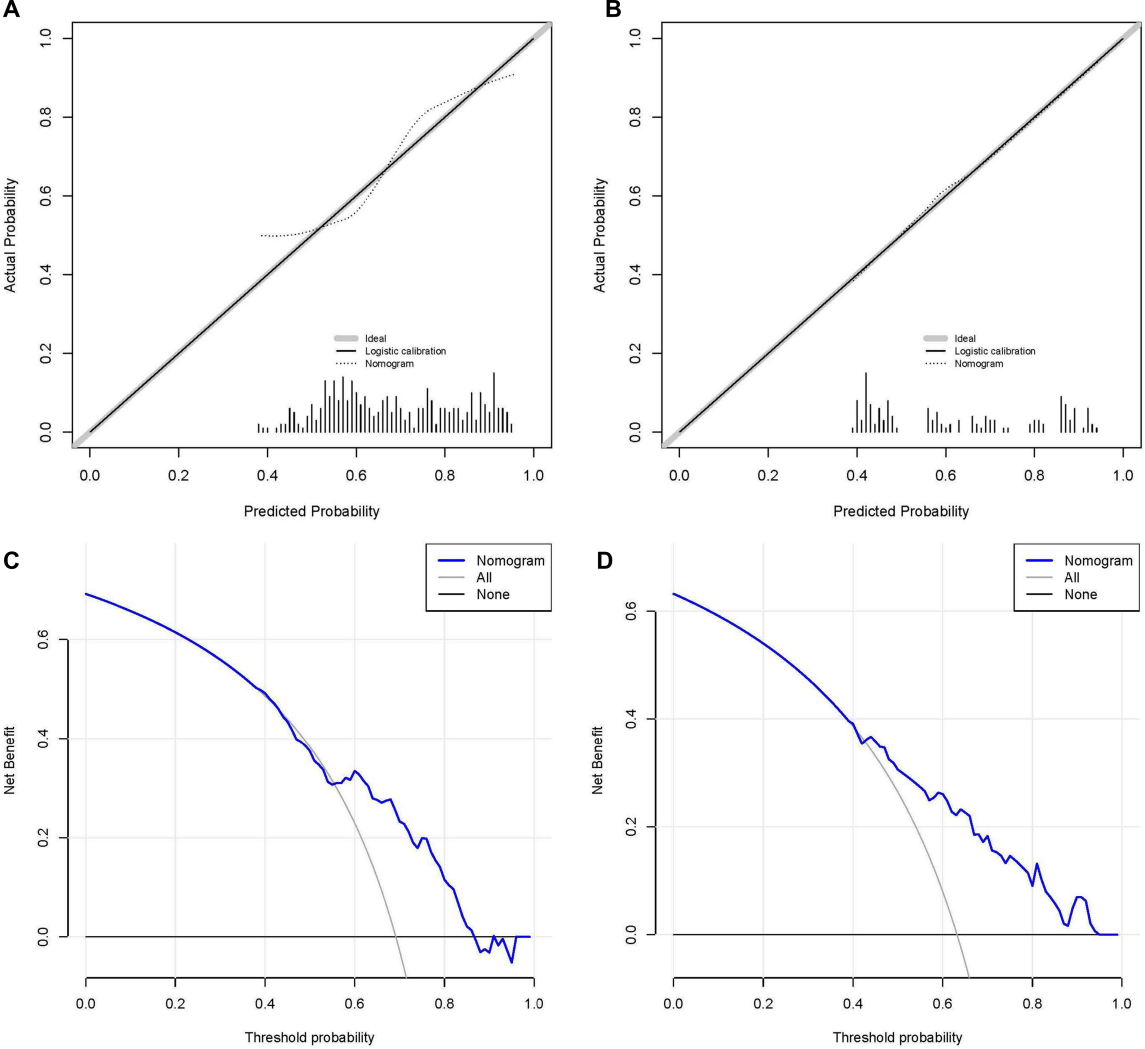
Calibration and decision curve analysis. Calibration curves of the nomogram in the training set (**A**) and the validation set (**B**), respectively. The nomogram's decision curve analysis in the training set (**C**) and validation set (**D**), respectively.

**Figure 6:**
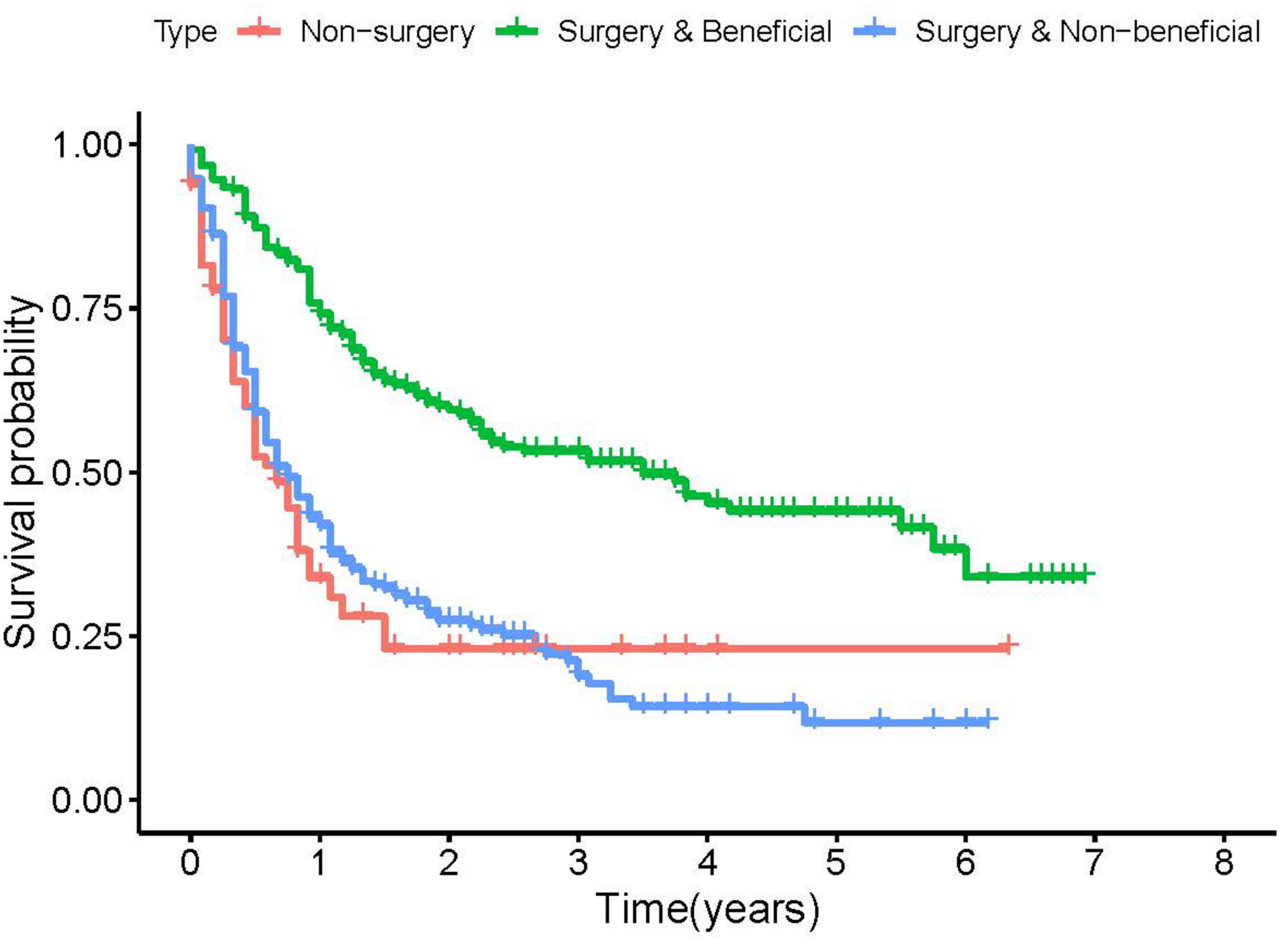
Kaplan-Meier plot to differentiate beneficial groups according to our model.

We established the ROC curves of the training set and the validation set (see Figure 4). The AUC of the nomogram was 0.727 in the training set and 0.755 in the validation set. At the same time, the calibration curves of the training set and the validation set reflected the robust calibration characteristics of the nomogram (Figures 5A,B). DCA indicated that the nomogram could be an excellent predictive model to identify stage IV CM patients suitable for PSR (Figures 5C,D). To further verify the discriminatory ability of the nomogram, we performed K-M analysis and log-rank test (Figure 6). The results showed that the prognosis was more in the beneficial-surgical group than in the non-beneficial-surgical group (*p*-values < 0.001) or the non-surgical group (*p*-values < 0.001). However, there was no difference between the non-beneficial & surgical group and the non-surgical group (*p*-values = 0.489). Based on the validation of the effectiveness of the nomogram, we established a web-based nomogram for further clinical promotion and application (https://zhehongli.shinyapps.io/skcm/).

### Clinical use of the web-based nomogram

**Figure 7:**
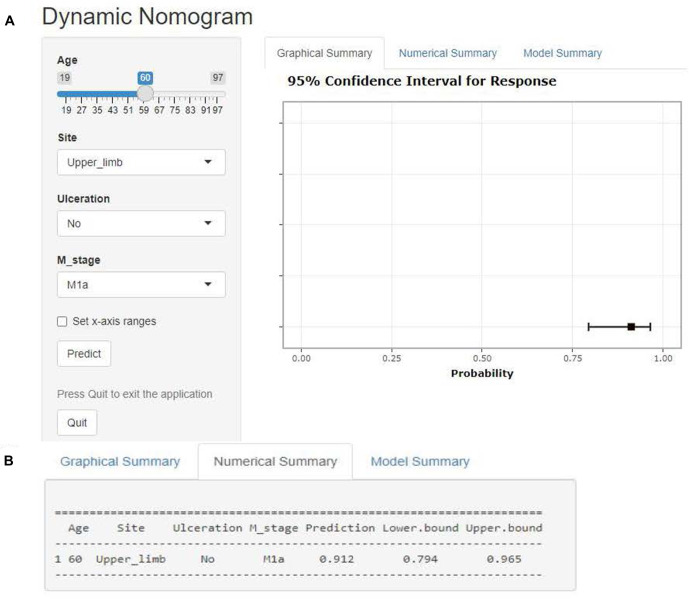
Web-based nomogram. Illustrate the web-based nomogram through an example. A stage IV CM patient has clinicopathological features: 60 years old, stage M of M1a, primary tumor location in the upper limb, and no ulceration at the primary site. Patient characteristics are shown on the left side of the network nomogram (Figure 5A left). The graphical summary (Figure 5A right) and the numerical summary (Figure 5B) show the probability line and the exact numerical value of the benefit (probability of surgical benefit of primary focus = 0.912, 95% CI = 0.794–0.965), respectively.

The operation interface of the web-based nomogram was shown in Figure 7A. We introduced the use of the web-based nomogram by way of an example. For example, a stage IV CM patient had clinicopathological features: 60 years old, stage M of M1a, primary tumor location in the upper limb, and no ulceration at the primary site. Patient characteristics were shown on the left side of the network nomogram (Figure 7A left). The graphical summary (Figure 7A right) and the numerical summary (Figure 7B) showed the probability line and the exact numerical value of the benefit (probability of surgical benefit of primary focus = 0.912, 95% CI = 0.794–0.965), respectively. Therefore, according to the conclusion of the web-based nomogram, this patient could benefit from PSR.

## Discussion

CM is a highly malignant tumor originating from melanocytes, which can develop in different tissues and organs such as skin, extremities, mucous membranes, and oculocutaneous membranes, etc (15). The prognosis of CM patients is not good due to its high degree of aggressiveness and metastatic nature (16). With the introduction of targeted therapies and immune checkpoint inhibitors, the survival of patients with advanced melanoma has improved (17, 18). Currently, it is controversial whether PSR should be performed on stage IV CM patients with a primary diagnosis. Many surgeons do not recommend local surgery for stage IV CM patients because the survival time for those is much lower than for patients with stages I-III (9, 10). However, previous retrospective studies have suggested a different perspective: PSR for metastatic CM improves patient prognosis (13, 19). Not all stage IV SCKM patients are suitable for PSR due to individual differences and particularities. In the era of precision therapy, determining the patient's benefit has tremendous significance for the prognosis of stage IV patients. The indications for PSR still need to be clarified due to the lack of relevant studies. Validating the premise that PSR can be beneficial for stage IV CM patients, our study was a pioneering effort to find those patients who are best suited for PSR. To our knowledge, this is the first study to identify the best candidates for PSR in stage IV SCKM patients.

We found that the surgical group's median CSS time was more prolonged (CSS: 17 months vs. 8 months, *p*-value <0.001). This conclusion further confirmed the necessity of PSR and corroborated Zhang et al. and Tauceri et al. (13, 19). Then, we further divided the patients in the surgical group into surgical benefit and surgical non-benefit groups using the median CSS time (8 months) of the non-surgical group. Finally, we used Cox regression analysis to identify independent prognostic factors and logistic models to construct a nomogram. After such a screening process, stage IV CM patients who were genuinely suitable for PSR were identified. In addition, we built a web-based nomogram to find the best surgical target. Meanwhile, the validation of the nomogram confirmed the excellent predictive performance of our model.

In the nomogram, younger age was one of the essential factors in the benefit from PSR. Older age was associated with worse outcomes for stage IV CM patients who underwent either primary or metastatic surgery (20, 21). This finding suggests that the patient's condition is critical to the traumatic impact of the surgery and the postoperative recovery. In addition, we have also noticed that patients with M1a (skin or subcutaneous metastasis, or distant lymph node metastasis) and M1b (lung metastasis) can benefit from PSR. However, the nomogram concluded that M1c patients were not recommended to undergo surgery, which may be closely related to the occurrence of the central nervous system (CNS) metastasis and/or increased lactate dehydrogenase (LDH) in M1c stage melanoma. LDH is recognized as one of the vital tumor prognostic markers, and its high expression often indicates poor prognosis (22). On the other hand, once melanoma is diagnosed with central nervous system metastasis, its prognosis is abysmal (median OS is only 4 months) (23). The revision of the 8th edition of the AJCC staging guidelines designates CNS metastasis as M1d, further reflecting that the OS of patients with CNS metastases is generally worse (4). Therefore, we have reason to believe that patients with M1c and M1d (8th edition of the AJCC staging guidelines) are not suitable for PSR. A retrospective study by Tas F et al. showed that five-year survival was lower in ulcerated melanoma than in non-ulcerated melanoma (55.3% vs. 81.5%, *p < *0.001) (24). Ulceration status is defined based on histopathologic examination of the absence of the complete epidermal allodermis over any part of the primary tumor with associated host response, and both the seventh and eighth editions of the AJCC staging guidelines consider ulceration as an additional T-category criterion (4, 25). Previous studies have shown that both breslow tumor thickness and ulceration are independent prognostic factors for OS in CM patients; therefore, we discussed T-staging into two variables: Breslow tumor thickness (T1, T2, T3, and T4) and ulceration (Yes and No) during the study (26, 27). Moreover, our nomogram model suggested that stage IV CM patients without ulceration were better candidates for surgery for the first time. Previous studies have demonstrated that the location of the primary tumor is an important prognostic factor, and whose primary site is the head and neck having a worse prognosis than CM originating from other sites (28, 29). Our study further deduces on this basis that stage IV CM patients whose primary site is the head and neck are more suitable for surgery than other primary sites.

Previous studies have demonstrated that PSR can extend survival time in patients with metastatic cancers that have been screened for, including non-small cell lung cancer (30, 31), breast cancer (32–34), kidney cancer (35), and colorectal cancer (36, 37). Firstly, symptomatic occupancy consequences are mitigated by surgical resection of primary site tumors. Secondly, tumor excision is helpful for confirming the diagnosis and determining the best course of treatment. Thirdly, PSR in metastatic cancer can prevent tumor-related complications and prolong survival time, but it is also associated with an increased chance of perioperative death (38). By extension, we deduced that before making specific judgments, the advantages and disadvantages of PSR for patients with stage IV CM must be thoroughly evaluated. Our research was carried out to identify the stage IV CM patients who would benefit from PRS. In our study, K-M plots were used to differentiate the beneficiary groups and showed that nomogram-screened patients suitable for surgery had a longer median survival time, with a statistically significant difference. Our study suggested that not all stage IV CM patients were suitable for PSR, that only specific patients will benefit from PSR, and that the potential benefit will vary depending on the characteristics of CM patients.

Some limitations of this study should be noted. Firstly, the lack of unknown information in the SEER database may have produced selection bias during data screening. Secondly, the site of distant metastasis is critical to the prognostic impact of melanoma (e.g., brain, lung, liver, bone, etc.), but there is a lack of relevant data for patients whose melanoma was diagnosed earlier than 2010. Thirdly, this is a retrospective analysis of the SEER database. We do not know the relationship between the quality of survival of CM patients with stage IV and other indicators that may impact prognosis (e.g., targeted therapy, immunotherapy, supportive care).

## Conclusions

Based on the confirmation that PSR benefits stage IV CM patients, we propose a new method to screen patients who would truly benefit from PSR. Our study suggested that not all stage IV CM patients were suitable for PSR, that only specific patients will benefit from PSR, and that the potential benefit will vary depending on the characteristics of CM patients. It should be noted that in stage IV CM patients, the younger the age, no ulceration, location in the head and neck, and non-M1c (M1a or M1b) patients will likely benefit from PSR. Meanwhile, we develop a dynamic nomogram (web-based nomogram, https://zhehongli.shinyapps.io/skcm/) based on a static nomogram with good predictive efficacy, achieving good clinical dissemination and application.

## Data Availability

The original contributions presented in the study are included in the article/Supplementary Material, further inquiries can be directed to the corresponding author/s.
